# Automatic Detection of Gaze and Body Orientation in Elementary School Classrooms

**DOI:** 10.3389/frobt.2021.729832

**Published:** 2021-09-01

**Authors:** Roberto Araya, Jorge Sossa-Rivera

**Affiliations:** Institute of Education, Universidad de Chile, Santiago, Chile

**Keywords:** gaze detection, body orientation detection, non-verbal behavior, teaching practices, student attention

## Abstract

Detecting the direction of the gaze and orientation of the body of both teacher and students is essential to estimate who is paying attention to whom. It also provides vital clues for understanding their unconscious, non-verbal behavior. These are called “honest signals” since they are unconscious subtle patterns in our interaction with other people that help reveal the focus of our attention. Inside the classroom, they provide important clues about teaching practices and students' responses to different conscious and unconscious teaching strategies. Scanning this non-verbal behavior in the classroom can provide important feedback to the teacher in order for them to improve their teaching practices. This type of analysis usually requires sophisticated eye-tracking equipment, motion sensors, or multiple cameras. However, for this to be a useful tool in the teacher's daily practice, an alternative must be found using only a smartphone. A smartphone is the only instrument that a teacher always has at their disposal and is nowadays considered truly ubiquitous. Our study looks at data from a group of first-grade classrooms. We show how video recordings on a teacher's smartphone can be used in order to estimate the direction of the teacher and students’ gaze, as well as their body orientation. Using the output from the OpenPose software, we run Machine Learning (ML) algorithms to train an estimator to recognize the direction of the students’ gaze and body orientation. We found that the level of accuracy achieved is comparable to that of human observers watching frames from the videos. The mean square errors (RMSE) of the predicted pitch and yaw angles for head and body directions are on average 11% lower than the RMSE between human annotators. However, our solution is much faster, avoids the tedium of doing it manually, and makes it possible to design solutions that give the teacher feedback as soon as they finish the class.

## Introduction

Educational researchers have collected information on teacher and student classroom behavior for more than a century. In 1912, Stevens counted the number of questions asked by the teacher per unit of time and the proportion of words spoken by the teacher compared with words spoken by the students (Stevens, 1912). In 1946, statistical information on different teacher practices in the classroom was collected via other methods such as filming individual teachers in action (National Education Association, 1946). This type of information is necessary for understanding the teaching practices that actually occur in the classroom. With the help of technology, it is increasingly possible to record information at a more granular level and analyze it more deeply. One of the first studies using videos to compare strategies across countries was the Third International Mathematics and Science Study TIMSS 1999 Video Study and its follow-up and expansion TIMSS 1999 (TIMSS, 1999) Video Study. Many other studies based on video lessons have been conducted since then. For example, transcriptions of slices from 710 videos of mathematics lessons taught by different teachers (Araya and Dartnell, 2008) revealed several insights, such as very little autonomous student participation, teachers neither presenting nor discussing any proofs, no use of information technology, almost no use of textbooks, and almost no explicit use of metaphors or analogies. Using the presence/absence of 8 categories of contents and 12 categories of teacher practices, those 710 videos were then rated by 4 human coders. Automatic classifiers were then trained with a support vector machine. For each human rater, the classifier trained with their data obtained a better level of agreement than the level of agreement between human raters (Araya et al., 2012).

One important challenge is that much of the teacher and students’ behavior is unconscious. Their unconscious interactions are a powerful tool that can help diagnose and then potentially improve teaching and learning practices. This unconscious behavior includes both verbal and non-verbal behavior. Both reveal interesting insights into what is happening in the classroom. In particular, the acoustic and linguistic features of their speech, and pattern of their gaze and their body orientation are “honest signals” (Pentland, 2010) that tell highly communicational information. These signals may have a significant impact on student attention and learning. This is information that teachers and students are constantly transmitting; immediately creating a chain of conscious and unconscious responses. However, teachers and students are not fully aware of most of this cascade of communication signals and the reactions they generate. This phenomenon can therefore not be investigated using interviews or questionnaires.

For example, acoustic features and ML models have recently been proposed as promising tools for analyzing lessons (Owens et al., 2017; Cosbey et al., 2019). Acoustic patterns, in both time and spectral domains, are related to the teacher’s pedagogical practices. They can predict when the teacher is lecturing, guiding or focusing on administrative tasks during the lesson (Schlotterbeck et al., 2021a). If we add the transcriptions to the acoustic features, then the accuracy of the predictions for the presence of these teaching practices improves, achieving over 88% accuracy and 92% AUC (Schlotterbeck et al., 2021b). It is important to note that not everything spoken and then transcribed is conscious (Pennebaker, 2011). There is a lot of information contained in the unconscious choice of connectors, prepositions and pronouns. Furthermore, it is not only what is said but also how it is said. For example, the words the teacher uses in questions have an effect on student responses. A limited number of keywords present in the question has impact on the length of the students’ answers (Araya et al., 2018). Words such as "explain" elicit longer written responses among fourth grade students (Araya and Aljovin, 2017).

In this work we study non-verbal signals. There is a wide variety of unconscious non-verbal information that can be analyzed. Gestures; positions of arms and legs; and movements of the eyebrows, mouth and shoulders provide a lot of relevant information (Collett, 2003). Eye contact is very important for team work, as well as being a basic nonverbal strategy in teaching (Johnson et al., 1994). However, in some contexts body orientation can have more of an impact. For example, a physician pointing their body toward the patient is sometimes more critical than making eye contact (Robinson, 2006). Head and body orientation are critical for communication among humans. Young children begin to carry out actions with others using joint visual attention at around 9 months old (Tomasello, 2014). However, communication gaze and body orientation are also critical for non-human animals (Davidson and Clayton, 2016). Terrestrial predators often send an “honest signal” (Bradbury and Vehrencamp, 1998) suggesting their subsequent course of action (Book and Freeberg, 2015). These signals not only reveal what they are going to do, but also in which direction they are going to move. Such signals are of great adaptive benefit for avoiding predators and for interacting with group members. For example, African elephants signal significantly less when the experimenter’s body is pointing away from the elephant (Smet and Byrne, 2014). It therefore appears that there is a strong connection between the importance of visual attention and body orientation for effective communication. Moreover, body orientation may be a more reliable signal of a predator’s intentions as it is more difficult to fake. Gaze is very easy to change and can be used to deceive prey or predators.

While gazes can be recorded with great precision using eye-trackers (Jarodzka et al., 2021), these devices are large, expensive, and not suitable for sustained use in classrooms. However, there is a new technology involving mobile eye-trackers that makes it possible to investigate teacher gaze in the classroom (McIntyre et al., 2019). While very powerful, this technology is still too expensive for regular use in the classroom by teachers. Another possibility is to use several cameras to later identify gazes (Ahuja et al., 2021) or to use stereo cameras (Abughalieh and Alawneh, 2021). However, this type of equipment is also complex and is not widely-available in schools. A third possibility is to use mini cameras (costing USD 50 or less) mounted on eye-glasses. These cameras show the first-person perspective, making it easy to accurately code who is looking at whom. Some of this first-person information helps understand the focus of the students’ attention. For example, first-person video recordings, obtained from micro cameras mounted on fourth graders’ eye-glasses, reveal different gaze patterns between groups according to gender, subject, student grade point average (GPA), sociometric scale and time of day (Araya et al., 2015). After 40 min of class, the gaze of low GPA students towards the teacher decreases much more than with high GPA students. Popular students, high GPA students, attractive boys, and girls without much upper body strength all receive significantly more gazes from peers throughout a class (Araya and Hernández, 2016). Furthermore, fourth graders gaze at the teacher lasts 50% longer when the teacher is gesturing. The data also revealed different effect sizes for gender, subject matter, and student GPA (Farsani et al., 2020; Hernández Correa., 2020). In particular, the effect of teacher gesturing on students with a low GPA is higher than on students with a high GPA. The teacher pointing their body toward the student attracts a student’s attention more in STEM classes than in other subjects (Araya and Farsani, 2020). We also found that this effect is greater among boys than girls, and that it is particularly evident for certain distances between the teacher and the student. These patterns are practically impossible to detect through surveys. Video analysis of footage from mini cameras mounted on eye-glasses is a powerful tool for teachers. This is because it can help them reflect on their strategies, as well as the collective social impact of their unconscious nonverbal behavior in class.

Although these cameras are cheap, it is still impractical for the teacher to use them on a regular basis. Moreover, manual analysis of the recordings can present several difficulties. One of the main problems is the slowness of the analysis. To encode using any of the established protocols, it is estimated that 4 h of coding are required for each hour of recording (Li et al., 2020). Another difficulty is the dependence on the encoder. For this reason, there must be a set of overlapping segments. These can then be used to measure the degree of agreement between coders (Tong et al., 2020). This makes the process even slower. Additionally, there is the self-consistency issue with each encoder. With boredom and fatigue, coders can gradually start to change their criteria without realizing it. Some of this inconsistency can be partially controlled with observer training programs. However, some programs require several days of intensive training before observations take place in the classroom. This slows down the entire procedure even more.

In this paper, we study the use of technology-independent devices to record and analyze the gaze and body orientation of teachers and students. Thanks to advances in technology, estimators of gaze and body orientation can now be developed without the need for any specialized external devices, such as the Microsoft Kinect One depth camera, or having students and teachers wear devices like accelerometers, mobile eye-trackers or other wearable devices. One simple solution is to use multiple cameras, and then to calculate the gazes using synchronized recordings (Ahuja et al., 2021). However, this solution still requires multiple cameras and their synchronization. Therefore, a more practical alternative must be found. In this paper, we study the possibility of using just the teacher's smartphone. This device is already ubiquitous across most of the world. Even in developing countries, teachers already have smartphones. This makes the solution very practical and truly scalable. It can also be implemented more easily as it does not involve acquiring or implementing any specific instruments.

Although there are a number of algorithms to determine the orientation of people using only one camera (Moreno-Noguer, 2016; de Paiva et al., 2020; Wu et al., 2020), they have several limitations for use in the classroom. They are focused on pedestrian body orientation and other situations that are of interest to autonomous vehicles. For example, they estimate the orientation of the body as an angle in the ground plane (Wu et al., 2020). Furthermore, they are not trained using elementary school classroom databases, where most of the students are seated and with strong occlusion between them and with classroom furniture. Similarly, they do not seek to determine the yaw and pitch of the head and body orientation. They do not integrate gaze with body orientation. On the other hand, (Chen and Gerritsen, 2021) use video from a single and wide-angle camera, but the camera is at a fixed location and it is at the front of the classroom. Additionally, the camera recorded university students and did not record the teacher. A total of 22 sessions taught by the same teacher were recorded. The authors also use OpenPose software. However, they use it to solve a classification problem to discriminate between two types of class conditions, and not to estimate head and body directions in a continuous-time.

**FIGURE 1:**
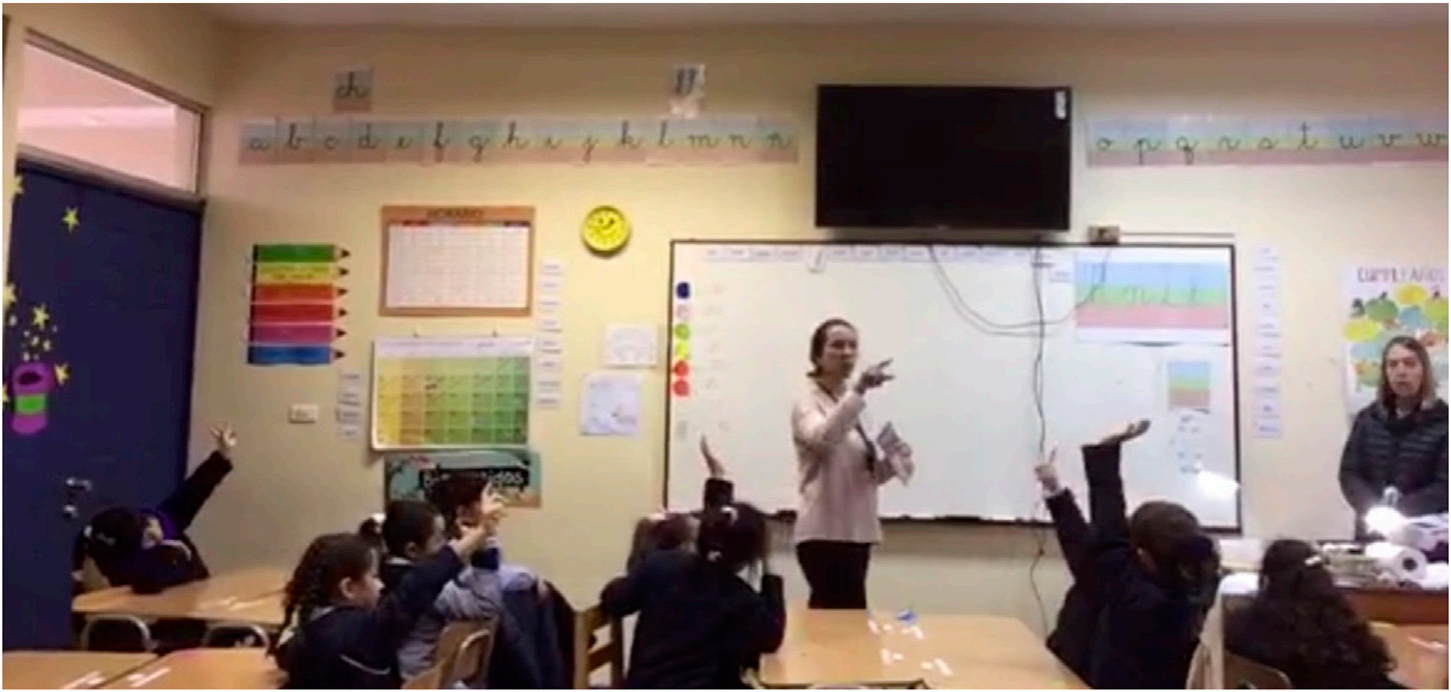
Typical frame of a video obtained with a smartphone placed at the back of the classroom.

Additionally, due to privacy concerns, some teachers prefer to avoid recording students’ faces as much as possible. We are therefore interested in exploring how the estimation of gaze and body orientation can be obtained using video recordings from a single smartphone located at the back of the classroom. By doing so, most of the scenes do not show the students’ faces (Figure 1). Moreover, teachers locate their smartphone in different locations, since they use their smartphone during the breaks for personal use, and the tripod used is a small portable tripod that they carry with their smartphone. Moreover, some teachers use a small tripod that rotates following the teacher, such as the Swivl device. Thus, the camera is not a fixed camera. To the best of our knowledge, this paper is the first that includes these restrictions, which are critical to facilitate use by teachers.

As we cannot count on having information from the students’ eyes and pupils, head orientation is instead used as a proxy for gaze. Our research question therefore asks:

To what extent can recordings from a smartphone placed at the back of the classroom be used to estimate the head and body orientations of the teacher and students at each moment of the lesson?

The organization of the paper begins with the description of the OpenPose software, and how it can be used to estimate the direction of the head and body. We propose some heuristics as baselines. Next, we describe the web environment where four raters manually annotated their estimation of the head and body directions in a sample of images of students and teachers obtained from frames of the recorded video. Using these annotations we train estimators with ML algorithms. Next, we calculate the difference (RMSE) between the estimates of the algorithms with the estimates of the human annotators. Finally, we compare these differences with the differences (RMSE) in a subset of the same images between human annotators.

## Materials and Methods

**FIGURE 2:**
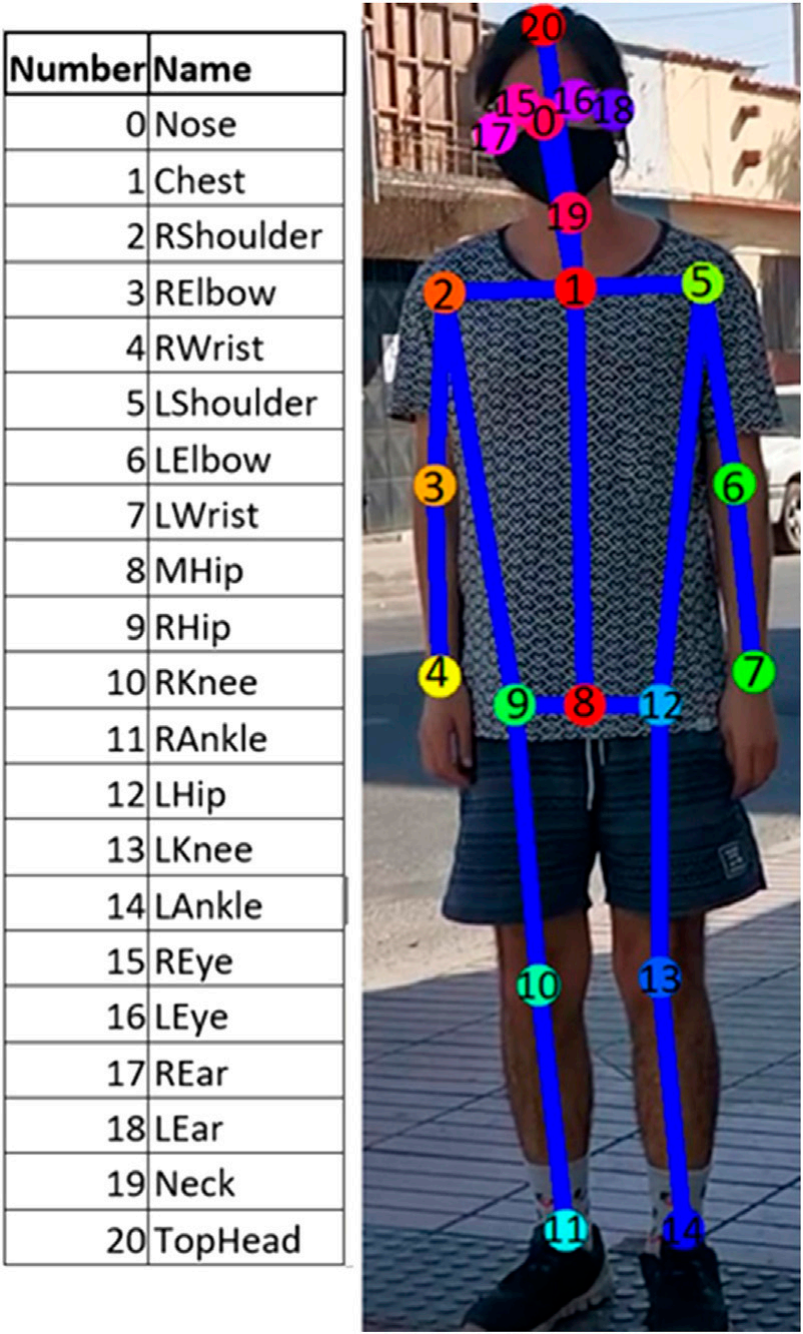
Key-points detected by OpenPose and their numeric code.

Similarly to (Ahuja et al., 2019; de Paiva et al., 2020), we use the OpenPose software for this purpose. This is a real-time multi-person system that jointly detects human body key-points on single images (Cao et al., 2017). For each video frame, OpenPose provides 21 body key-points for each subject in the classroom (Figure 2).

**FIGURE 3:**
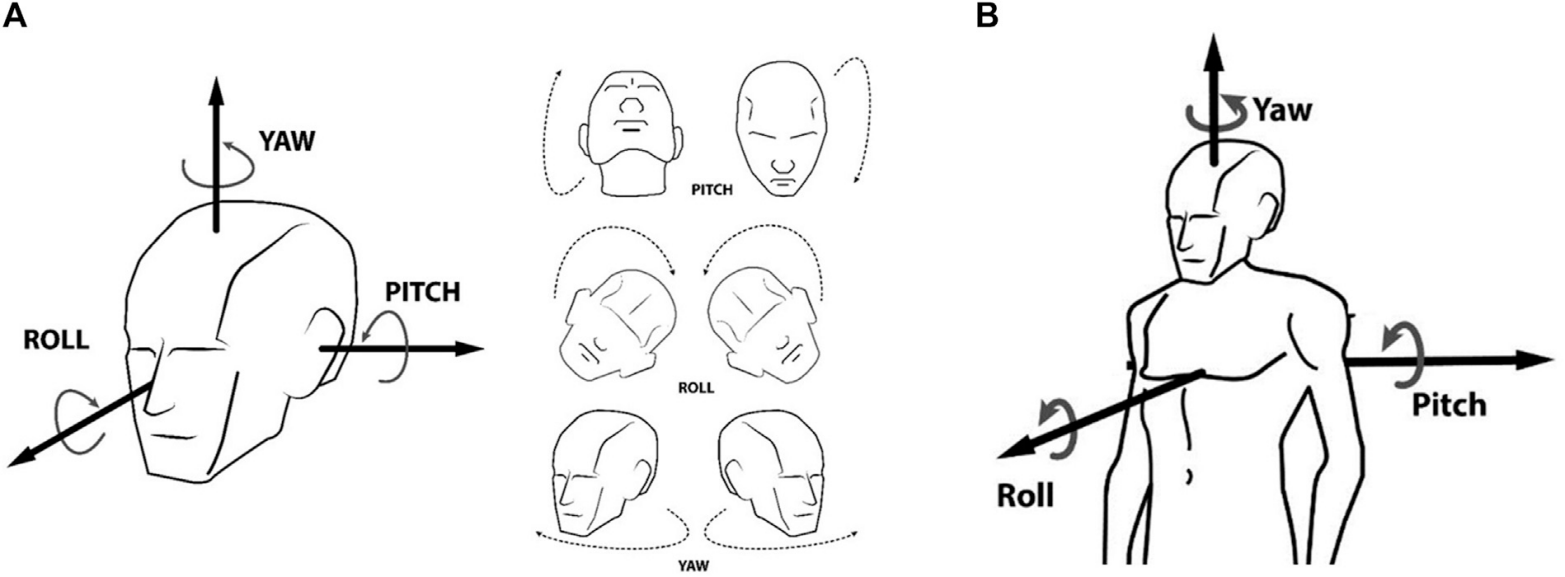
Yaw and pitch angles of the head, and yaw and pitch angles of the body at chest height. **(A)**: Yaw and pitch angles of the head. **(B)**: Yaw and pitch angles of the body at chest height.

Our goal is to find four angles at each moment and for each subject. These angles are the yaw and pitch of the head, as well as the yaw and pitch of the body (Figure 3). For this purpose, we use the output from OpenPose for each video frame.

Our methodology is first to find some basic heuristics. We then compare the agreement of these heuristics with manual estimations of these angles made by human annotators. Following this, we then use ML to find better estimators with higher levels of agreement with human estimations. In this case, we first use decision trees to gain some insights, before then using the random forest algorithm.

Using different heuristics, we define baseline estimators for the four angles: yaw and pitch of the head, and yaw and pitch of the body (Figure 3). However, these baselines are not designed based on real data. Since videos from smartphones are not professional nor high quality, it is important to consider this limitation in the design process. The idea is to find estimators that adjust to the real situation in the classroom. It is critical to use videos captured with typical smartphones, recorded from the back of the room, and with the output that OpenPose provides under these restrictive conditions. Using ML we explore estimators that are robust to noisy data.

For ML we use the output of OpenPose and manual codification of the yaw and pitch angles of the head and body made by human annotators. We use videos from 4 of the 60-min sessions recorded using smartphones. These correspond to 4 different first grade classes. The camera was located at the back of the classroom in three classes (Figure 1) and at the front in the other. From these videos, we obtained a sample of 1,991 frames for gazes and 1,991 frames for body orientation. In each of these frames the teacher or a student are identified.

**FIGURE 4:**
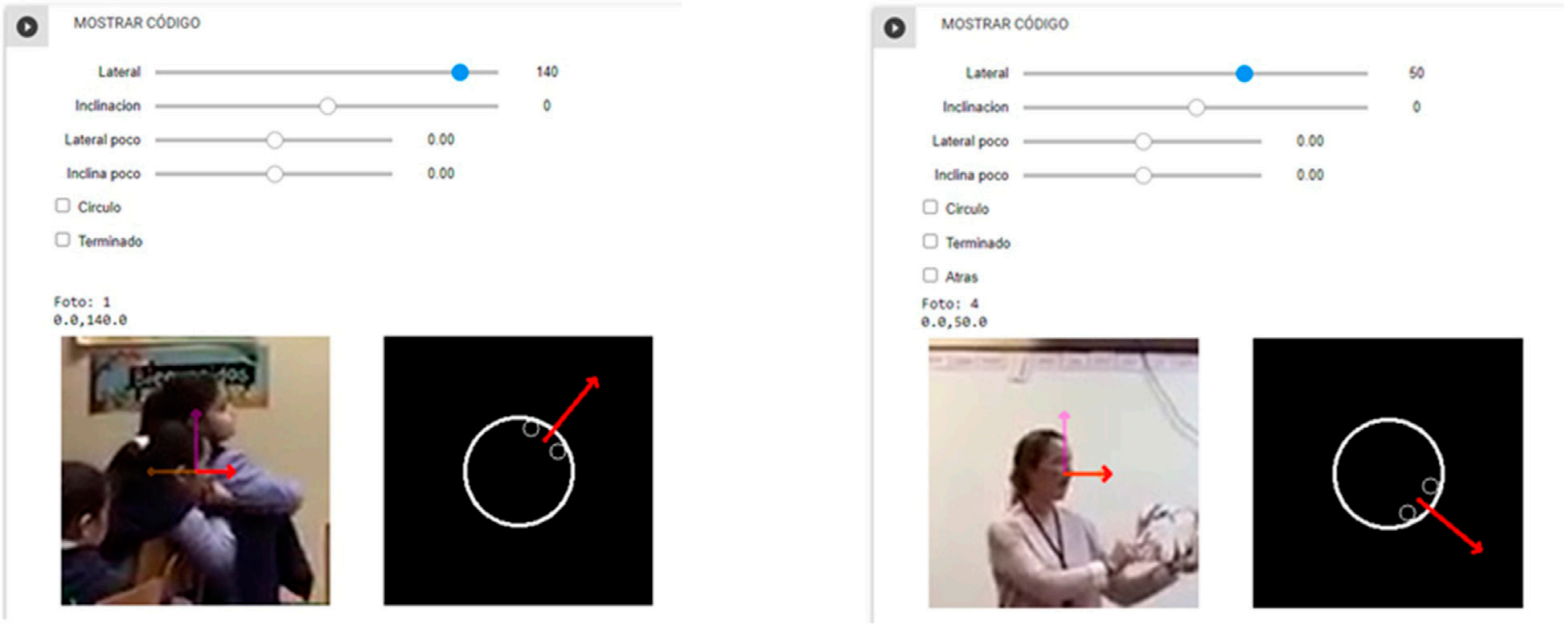
Screenshot of the annotator interface. Left, screenshot showing a student. Right, screenshot showing the teacher. In each case, the annotator has to adjust the red arrow moving the four bars at the top. The annotator has two view of the red arrow. One view is in the video frame. The other view is an iconic representation of the subject and the red arrow as it would be seen from above.

Four elementary school teachers annotated yaw and the pitch angles of the head (Figure 3A), and yaw and pitch angles of the body at chest height (Figure 3B). In order to collect this situated information, we built a web-based annotation interface. The annotation system selects a subject and the annotator has to manually annotate the angles. To do this task, a web interface shows a red arrow to represent the direction in which the subject may be pointing their head or body. The annotator then has to adjust the arrow. To move the arrow in the desired direction the annotator has 4 bars. The first 2 bars help to move the yaw and pitch. The other 2 bars help to move the yaw and pitch with a finer degree of tuning (Figure 4). To facilitate this task the annotator system provides two views. One view is a section of the screenshot of the frame from the video with the red arrow. The other view is from above. The latter is a schematic view. It helps the annotator to determine the correct directions. Once the annotator finishes adjusting the arrow, the system then calculates and saves the corresponding yaw and pitch angles in the database.

Following this method, we obtained training data comprising 1,991 annotated images for gazes with the corresponding pitch and yaw angles, and 1,991 annotated images for body orientation with the corresponding pitch and yaw angles. For each frame, we have all or part of the key-points for a particular subject (Figure 3). However, since the teacher and most of the students are often partially occluded by other students or furniture, they do not appear in full on the videos. Thus, OpenPose does not always give the position of any unseen parts of the body. Nevertheless, 47,900 variables were defined and computed using the available key-points. These variables include the positions x and y of each key-point, the angles with the horizon of the vector obtained from each pair of key-points, the length between every pair of key-points, ratios of all pairs of these lengths, proportions of these lengths with respect to the maximum extension inferred from a standard body model, and proportions of these lengths with respect to the maximum extension inferred from a body model generated using the components of the same person observed in the video.

We analyzed each of these variables and only retained those that had values for at least 20% of the sample. This procedure reduced the number of variables to 32,486 for the gaze data. The final gaze database therefore includes 32,486 columns and 1,991 rows. In the case of body orientation, the final number of variables was 32,262. Therefore the body orientation data base has 32,262 columns and 1,991 rows.

The baseline for the gaze yaw uses the proportion of the horizontal distance from the nose to the center of the face (defined by the neck axis) to the maximum distance reached in the video for the subject. The baseline for the gaze pitch uses the proportion of the difference in height between eye and nose to the maximum difference reached in the video for the subject. The baseline for the body orientation yaw uses the proportion of the shoulder´s length to the maximum of the shoulder´s length reached in the video for the subject. It also looks at whether or not the nose is visible in order to determine whether the movement is forward or backward. The baseline for the body orientation pitch uses the proportion of the length of the torso to the maximum reached in the video for the subject. All of these heuristics are appropriate under ideal conditions when the coordinates of the key-points are accurate. However, due to the quality of the videos, these key-point coordinates are very noisy.

In the search for better predictors of the four angles we turned to ML. From the sample of 1,991 gaze images of subjects obtained from 1,991 video frames, we separated 1,333 images for training and 658 images for testing. For each image we have the 32,486 variables. Similarly, from the 1,991 body orientation images, we separated 1,333 images for training and 658 for testing. For each body orientation image, we have 32,262 variables. We categorized the angles into various granularities in order to use automatic classifiers. We then used tools from scikit-learn Machine Learning in Python. We first used decision trees (https://scikit-learn.org/stable/modules/tree.html), a non-parametric supervised learning method which is simple to understand and interpret. We replaced missing values (NaNs) with an extreme value of the corresponding variable. We then applied the Minimal cost-Complexity Pruning algorithm (https://scikit-learn.org/stable/modules/tree.html, section 1.10.8) to obtain pruned trees. This is an algorithm used to prune a tree in order to avoid over-fitting. Finally, we then applied the Random Forest classifier (https://scikit-learn.org/stable/modules/generated/sklearn.ensemble.RandomForestClassifier.html), an algorithm that generates a number of decision tree classifiers on various sub-samples of the dataset and uses averaging to improve the predictive accuracy and control over-fitting. In each case, we calculated the corresponding root mean square error (RMSE).

**FIGURE 5:**
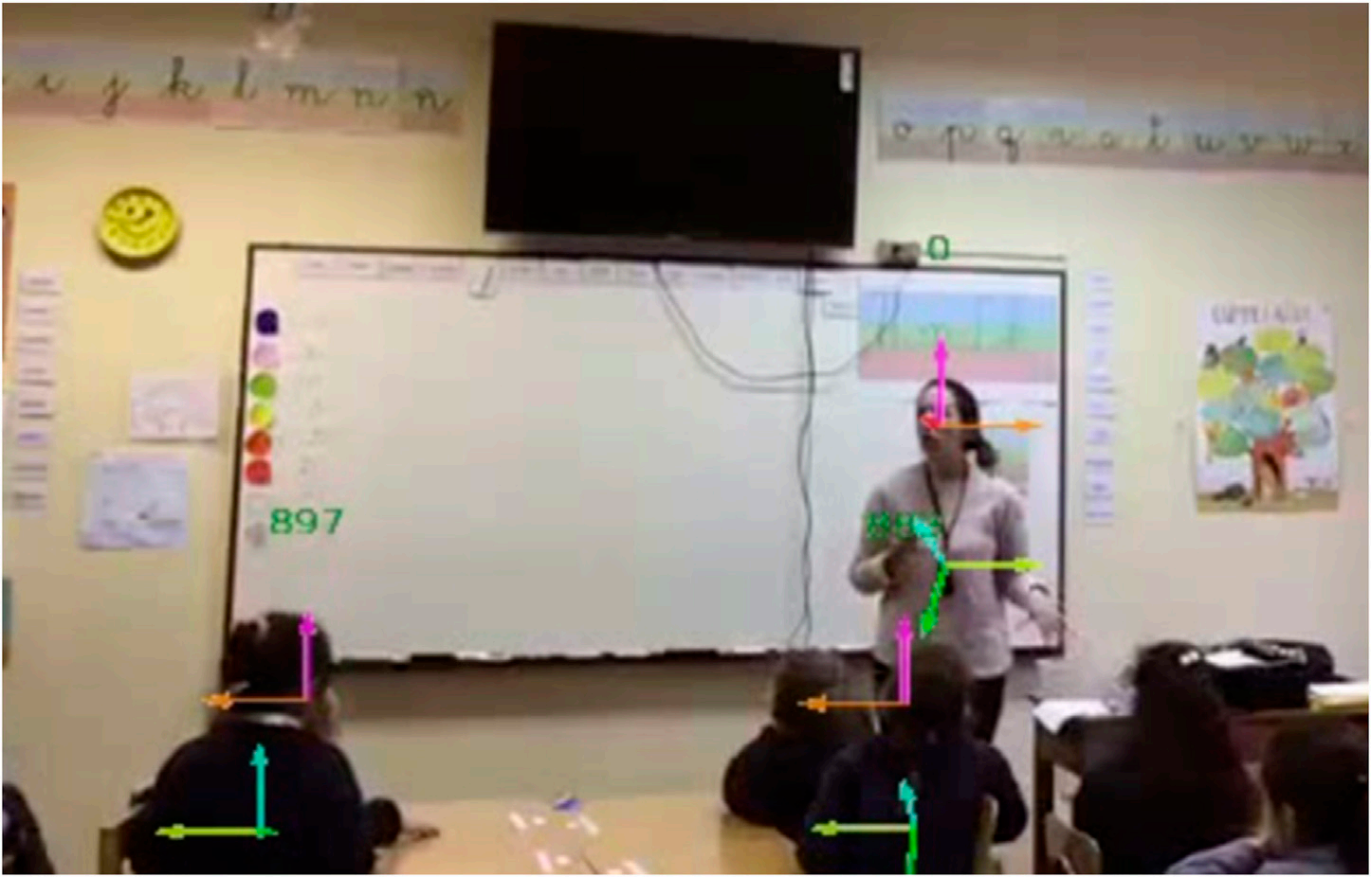
Screenshot of the output of one estimator for the teacher gaze and body orientation, and for some of the students. The numbers are identifiers of the subjects.

The output of this procedure generates vectors that are then superimposed on the videos (Figure 5). This allows the images to be reviewed quickly and to detect any instances in which the prediction needs to be improved.

**FIGURE 6:**
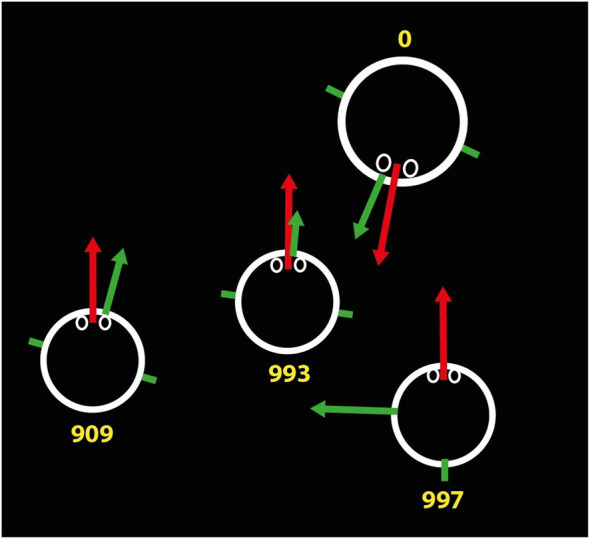
Representation of the teacher and students. The teacher is represented by the larger circle. Red arrows indicate head orientation, and green arrows indicate body orientation, with a vector normal to the chest. The shoulders are represented by two short green strokes.

Another view is a schematic from above, as shown in Figure 6, where in this case the teacher and three students are represented. Each one has the direction of the head (red arrow) and the direction of the body (green arrow) projected onto the floor plane. For example, in Figure 6 we can see a student whose head is turned (yaw) 90⁰ with respect to the body. In this schematic view, the lengths of the arrows provide information on the pitch angles. For example, in the case shown below, the teacher has a more inclined body (pitch) than head.

**FIGURE 7:**
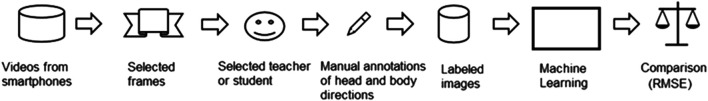
From videos recorded from the teachers' smartphones mounted on tripods behind the class, we randomly selected a sample of frames. Then, from each frame, we select an image of the teacher and another of a student. These images were used by four raters to manually annotate the angles of the head and body directions. In this way, we build a database of annotated images. In a training sample we ran ML programs to learn the patterns, and in an independent test sample, we compared the predictions with the annotations.

A summary of the procedure is shown in Figure 7. We used four videos, 60 min each, of first-grade classes, taught by their four respective teachers. From these videos, we randomly selected 1,991 frames for head direction and 1,991 frames for body direction. From these frames, we randomly selected the image of a student or a teacher. Half of the images were of teachers and the other half of students. Then 4 annotators recorded the yaw and pitch angles using the web interface described above. With the images of three classes, we trained various ML methods to estimate the angles. Then we tested the learned algorithms on the images of the remaining class. We compared the predictions of the algorithms with the manual annotations and calculated the respective RMSEs. We then compared those RMSEs with the RMSEs between the annotators.

## Results

To determine the agreement between the 4 annotating teachers with regards to the gaze pitch and the gaze yaw we used N = 310 images that were analyzed and annotated by the 4 teachers. As shown in the first row of Table 1, we obtained a RMSE between teachers of 13.58⁰ for the pitch, with a RMSE of 41.63⁰ for the yaw. For the body orientation pitch and yaw angles we used N = 309 images that were analyzed and annotated by the 4 teachers. In this case, we obtained a RMSE between teachers of 15.26⁰ for the pitch and 38.84⁰ for the yaw.

**TABLE 1:** RMSE (in degrees) for gaze and body orientation pitch and yaw angles, based on the estimations provided by the four teachers, and the respective 95% Confidence Intervals.

	**RMSE gaze pitch**	**RMSE gaze yaw**	**RMSE body orientation pitch**	**RMSE body orientation yaw**
Between teachers	13.58⁰	41.63⁰	12.91⁰	41.06⁰
CI Between teachers	[12.8, 15.0]	[39.1, 45.9]	[12.1, 14.2]	[38.5, 45.2]
Self	7.33⁰	36.96⁰	6.46⁰	19.62⁰
CI Self	[6.5, 8,3]	[32.6, 41.9]	[5.8, 7.4]	[17.5, 22.5]

Some images appeared twice at random times. Using these images, we calculated the average RMSE for the 4 teachers when comparing each teacher with themselves. This was done with N = 119 gaze images and N = 122 body orientation images for each teacher. This is a measure of self-consistency. As shown in the second row of Table 1, we obtained a RMSE of 7.33⁰ for gaze pitch and 36.86⁰ for gaze yaw. We also obtained a RMSE of 7.35⁰ for body orientation pitch and 17.36⁰ for body orientation yaw.

Table 2 shows the results of the baselines. The RMSEs are clearly higher than the RMSEs among teachers.

**TABLE 2:** Average of the RMSE (in degrees) of the baselines for the pitch and yaw of the gaze, and the pitch and yaw of the body orientation, with respect to the estimations of those angles provided by the teachers, and the respective 95% Confidence Intervals (CI). In general, the RMSE of the baselines is 10 degrees higher than the RMSE of the teachers.

	RMSE gaze pitch	RMSE gaze yaw	RMSE body orientation pitch	RMSE body orientation yaw
Baselines	23.76 ⁰	60.64⁰	35.88⁰	37.93⁰
CI	[22.3, 25.3]	[56.9, 64.4]	[33.7, 38.1]	[35.6, 40.3]

For the gaze pitch, we categorized the angle based on different granularities. When running decision trees, the best estimator for the gaze pitch achieved a RMSE of 14.29⁰ with the test data (Column 1, Table 3). For the pruned tree, a RMSE of 15.46⁰ was achieved using the test data. When using the random forest algorithm, a RMSE of 13.38⁰ was obtained using the test data. The result was obtained at the highest level of granularity of gaze pitch categorization. This RMSE is slightly lower than the RMSE between teachers.

**TABLE 3:** RMSE (in degrees) of the best estimators using decision trees, pruned trees, and random forest algorithms for gaze and for body orientation with the test data, and the respective 95% Confidence Intervals (CI).

	RMSE gaze pitch	RMSE gaze yaw	RMSE body orientation pitch	RMSE body orientation yaw
Decision Tree	14.29⁰	41.38⁰	13.00⁰	38.62⁰
CI Decision Tree	[13.4, 15.2]	[38.8, 44.0]	[12.2, 13.8]	[36.3, 41.0]
Pruned tree	15.46⁰	41.78⁰	11.85⁰	39.76⁰
CI Pruned tree	[14.5, 16.4]	[39.2, 44.4]	[11.1, 12.6]	[37.3, 42.3 ]
Random Forest	13.38⁰	35.47⁰	12.39⁰	31.84⁰
CI Radom Forest	[12.6, 14.2]	[33.3, 37.7]	[11.6, 13.2]	[29.9, 33.8]

We also categorized gaze yaw into different levels of granularity. We selected the granularity with the lowest RMSE. The random forest algorithm achieved the lowest RMSE (35.47⁰), which is lower than the RMSE between teachers (41.63⁰), and this difference is statistically significant at 95% confidence level. Moreover, the RMSE is even slightly lower than the average of the Self-consistency RMSEs (36.96⁰) (Column 2, Table 3).

We also categorized body orientation pitch into different levels of granularity. The corresponding RMSE for the best estimators across granularity levels and algorithms are shown in the third column of Table 3. The lowest RMSE for the test data was 11.85⁰, which was obtained with a pruned tree. This RMSE is lower than the RMSE between teachers (12.91⁰) (Column 3, Table 3).

Finally, we also categorized body orientation yaw into different levels of granularity. The best estimator across granularity levels and algorithms was obtained with the random forest algorithm. The RMSE in this case is 31.84⁰ (Column 4, Table 3). This is lower than the RMSE between teachers 41.06⁰, and this difference is statistically significant at 95% confidence level.

One of the variables used for training and testing was the location of the camera, i.e. at the front or back of the classroom. 75% of the images were captured from the camera on the smartphone located at the back of the classroom. However, examining all of the algorithms, we found that this variable is not included in any of the trees. The performance of the ML algorithm does therefore not depend on the location of the camera.

## Discussion

In order to provide the teacher with a practical tool for analyzing the non-verbal behavior in their teaching practices, the proposed solution must ensure several conditions. First, it must use the teacher's smartphone and avoid any additional equipment. This means that we have to consider low quality images with low resolution and with some indistinguishable parts of the body. Second, we have to consider video recordings from the back of the classroom. Third, in most situations, the students and teacher are partially occluded by other students and furniture. However, using a smartphone and avoiding other cameras or wearables makes the solution truly scalable. Fourth, based on privacy concerns, some teachers prefer to record from the back of the classrooms so as to avoid capturing the students’ faces. We therefore have to consider that the typical data will not show the students’ faces and will instead only show the teacher’s. Fifth, the proposed solution has to consider that from one session to another the teacher will locate her smartphone in different locations, since she will probably use her smartphone during the breaks for personal use, and the tripod will be a small portable tripod that she will carry with her smartphone. Moreover, some teachers use a small tripod that rotates following the teacher, such as the Swivl device. Thus, the camera is not located in a fixed position. Sixth, there are several factors that influence the quality of the videos´ images and the ease of detecting head and body directions. For example, the angle of the camera, light sources, and the brightness of the classroom. These factors affect the performance of the ML algorithms and also human annotators. In future work, it would be important to analyze how both the performance of the ML algorithms and the performance of human annotators depend on these factors. Most of these conditions are not considered in the solutions proposed in the literature. To the best of our knowledge, this paper is the first to meet all these conditions, which are necessary to facilitate use by teachers. In summary, a practical solution has to be based on the teacher's face and body, as well as the head and body of the students recorded mainly from behind. The challenge then is to be able to capture the non-verbal behavior of the students as well as that of the teacher under these restrictive conditions.

In our study, we used first grade classes recorded on a teacher's smartphone. We found that it is possible to estimate the direction of the teacher and students’ head and body orientation. Using the output from the OpenPose software, we ran ML algorithms to train an estimator of these directions. We found that the level of accuracy achieved is much better than several baseline estimators based on different heuristics. It is also comparable to the levels achieved by human observers watching frames of the videos. The mean square errors (RMSE) of the predicted pitch and yaw angles for head and body directions are on average 11% lower than the RMSE between human annotators. Moreover, the RMSE of the predicted yaws are statistically significant lower that the RMSE between teachers at a 95% confidence level. However, the solution based on ML is much faster, avoids the tedium of doing it manually, and makes it possible to design solutions that give the teacher feedback as soon as they finish the class. By doing so, our solution therefore provides a positive response to our research question.

A significant benefit of using OpenPose and saving data on gaze direction and body orientation is that it facilitates the storage of anonymous information. In this sense, it is not possible to determine with absolute certainty which student is providing just the gazes and body orientations. Another benefit is the reduction in the size of the information that is stored. A third benefit is the ability to subsequently perform very specific searches. For example, searching for precise moments when more than 20% of the students simultaneously turned their gaze towards the whiteboard or the teacher.

In the future we plan to tackle several challenges. First, it would be interesting to explore other machine learning algorithms and thus check whether the current errors can be improved. Second, gathering more accurate information on the four angles would also have a significant impact. This can be done with a scale model of the entire classroom. Another possibility is to properly equip a classroom in order to determine the angles with a high level of precision. Third, there is the need to develop heuristics and explore ML to determine the position of students and the teacher in the classroom. Fourth, another challenge is to improve the tracking of the individuals in the room. Fifth, it would be highly desirable to integrate the gaze and body directions with the positions, and with the teacher's speech obtained from their smartphone (Schlotterbeck et al., 2021a; Schlotterbeck et al., 2021b; Lämsä et al., 2021; Uribe et al., 2020; Altamirano et al., 2020). Sixth, it is necessary to develop a solution that integrates everything into a single platform that allows the teacher to review their class and receive a diagnosis of their teaching practices. Attention and body orientation detectors such as those proposed in this work are essential components to develop artificial agents that will observe, analyze and give feedback to the teacher, and improve Lesson Study methodologies (Araya, 2021). Seventh, it is necessary to conduct usability studies with teachers to determine what information to provide and how to represent it graphically. For example, in addition to total percentages of attendance in the session, (Araya and Hernández, 2016) incorporates a graph with an average timeline by groups of students, according to sex, grade point average, popularity, among others. Similar timelines can be useful for teachers.

A limitation of this work is that we do not calculate the direction of the gaze using the subjects’ pupils. Instead, we use the orientation of the head as a proxy. The direction of the head has been shown in empirical studies to be related to the student's performance, the proximity of the student to the teacher, the teacher's gesticulation, the student's fatigue during the session, and the time of day (Araya et al., 2016; Araya et al., 2020; Goldberg et al., 2021). These studies suggest that the direction of the head is a good proxy for the direction of gaze and attention. However, of more than 200 species of primates, the human is the only one with visible white sclera (Kobayashi and Kohshima, 2001). Experiments with 12-month-old children indicate that, unlike other primates, children pay more attention to the gaze of others than to the orientation of their heads (Tomasello, 2014). Therefore, in the future it would be important to examine with higher precision the difference between the gaze according to the subject’s pupils and the orientation of their head. This should be done in a classroom setting, in order to understand the frequency and impact of such differences.

## Data Availability

The datasets presented in this article are not readily available because the visual nature of our data and the ethical guidelines that this project follows don’t allow us to share the data set. Requests to access the datasets should be directed to roberto.araya.schulz@gmail.com.
